# Sequential Graft Limb Occlusion Following Endovascular Aneurysm Repair: A Case Report and Literature Review

**DOI:** 10.7759/cureus.60102

**Published:** 2024-05-11

**Authors:** Ioannis Kontes, Vangelis Bontinis, Alkis Bontinis, Vasiliki Manaki, Angeliki Chorti, Argyrios Giannopoulos, Kyriakos Ktenidis

**Affiliations:** 1 Department of Vascular Surgery, AHEPA (American Hellenic Educational and Progressive Association) University General Hospital, Aristotle University of Thessaloniki, Thessaloniki, GRC; 2 Department of Surgery, AHEPA (American Hellenic Educational and Progressive Association) University General Hospital, Aristotle University of Thessaloniki, Thessaloniki, GRC

**Keywords:** clopidogrel resistance, evar, sequential limb occlusion, iliac limb occlusion, graft limb occlusion

## Abstract

Limb graft occlusion (LGO) is a common complication that can occur after endovascular aneurysm repair (EVAR). There are many factors that can contribute to LGO, including patient-related variables, device-related considerations, and factors associated with the procedural technique. Patients with LGO may exhibit no symptoms, have intermittent claudication, or suffer from acute limb ischemia. In this manuscript, we present a case of a 64-year-old male who experienced sequential LGOs after EVAR accompanied by a comprehensive review of the pertinent literature.

## Introduction

Over the preceding two decades, endovascular aneurysm repair (EVAR) has predominantly supplanted open surgical repair as the primary modality for addressing abdominal aortic aneurysm (AAA) disease [1].

While EVAR has demonstrated superior peri-procedural morbidity and mortality outcomes, particularly benefiting elderly patients or those with significant co-morbidities, it is essential to note that EVAR is not exempt from complications. In particular, limb graft occlusion (LGO) represents a frequent complication manifesting following EVAR that sometimes may lead to complete endograft occlusion [2].

Multiple contributing factors to LGO exist, encompassing patient-related variables, device-related considerations, and factors associated with the procedural technique. Furthermore, individuals afflicted with LGO may present as asymptomatic, exhibit intermittent claudication, or manifest acute limb ischemia [3].

In accordance with the guidelines established by the European Society for Vascular Surgery (ESVS), in the presence of suspected LGO following EVAR, it is recommended that patients undergo computed tomography angiography (CTA) for the purpose of conclusively confirming the pathological condition [1]. Ultimately, treatment regimens diverge and are contingent upon the extent of the lesion and the symptomatic status of patients, encompassing a spectrum from conservative management to either endovascular or surgical repair.

Herewith, we present a case detailing sequential LGOs and ultimately a complete endograft thrombosis subsequent to EVAR, and a comprehensive review of the literature. Written informed consent has been obtained from the patient for publication of the case report and the accompanying images. We have the approval of the Ethics Committee of our institution for our study (ID No. 60087).

## Case presentation

A 64-year-old male diagnosed with paroxysmal atrial fibrillation (AF) under apixaban pharmacological therapy and a history of allergic reactions to β-lactamase antibiotics underwent elective EVAR for the treatment of infrarenal AAA characterized by multi-saccular morphology and a maximum diameter of 4.9 cm.

The anatomical characteristics of the lesion encompassed a reverse tapered infrarenal aortic neck with a diameter ranging from 2.3 cm to 3.1 cm and a length measuring 2.1 cm. Additionally, the distal aortic diameter was 1.8 cm; the right common iliac artery exhibited a diameter of 2.1 cm accompanied by a concomitant saccular aneurysm. The left common iliac artery demonstrated a diameter of 1.25 cm, while the external iliac artery (EIA) measured 0.8 cm in diameter on both sides. No significant angulation or tortuosity of the iliac vessels was documented.

The procedure was executed under local anesthesia via bilateral cut-down incisions. The patient received a TREO bifurcated stent graft (Terumo Aortic (US), Bolton Medical Inc., Sunrise, FL), comprising a 36 x 100 mm main body, a 15 x 100 mm left limb, and an 11 x 140 mm right limb, extending into the right external iliac artery (REIA).

The postoperative antithrombotic regimen consisted of aspirin administered once daily at a dosage of 100 mg, in conjunction with apixaban twice daily at a dose of 5 mg (the latter being a medication previously prescribed for AF). Promptly in the postoperative phase, subsequent to the administration of the initial aspirin dose, the patient exhibited a mild allergic reaction characterized by a whole-body rash. Consequently, aspirin was discontinued, and it was substituted with clopidogrel at a daily dosage of 75 mg. The patient was discharged five days after the procedure demonstrating full mobility and palpable femoral pulses.

**Figure 1:**
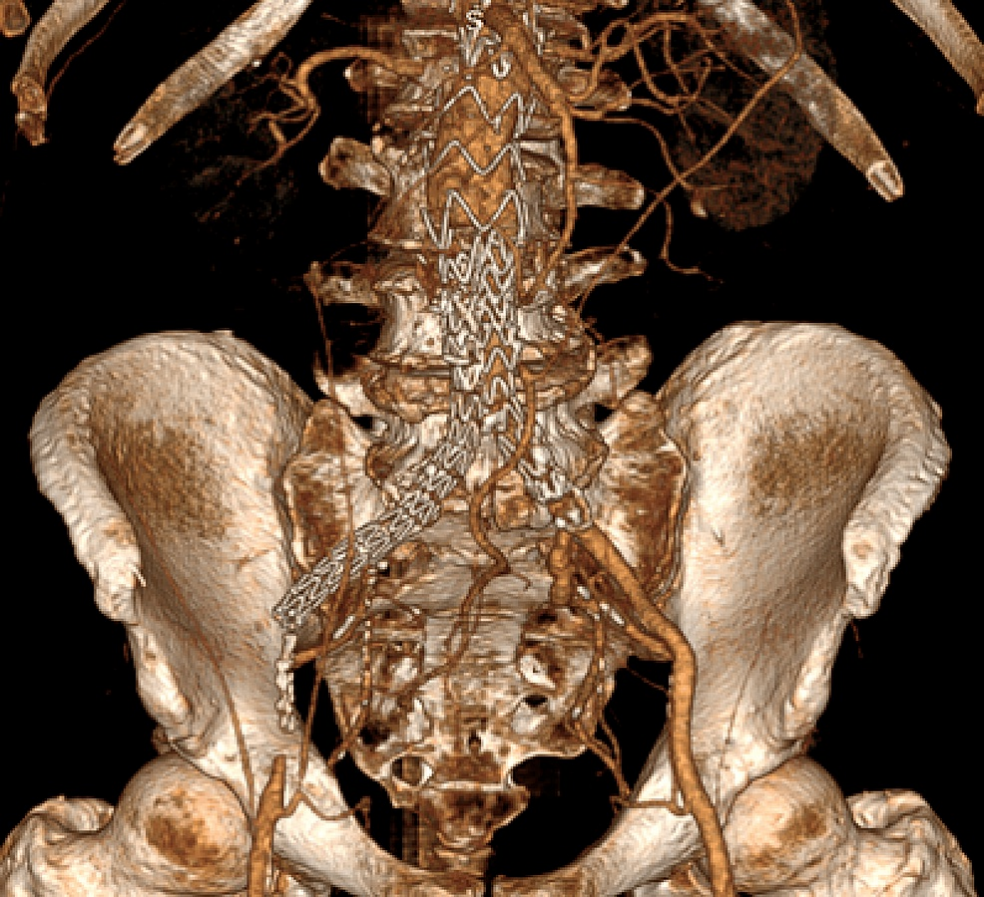
Right Limb Graft Occlusion First follow-up CT scan (3D reconstruction). Patent left iliac limb and occluded right iliac limb.

During the patient's monthly follow-up, he exhibited a mild generalized rash, which, according to his account, exacerbated subsequent to clopidogrel administration. Additionally, he reported symptoms of claudication affecting both lower extremities. During the clinical examination, pulses were conspicuously absent at the right femoral artery, whereas palpable pulses were discerned at the left femoral artery. Color duplex and CTA examinations confirmed the presence of right limb occlusion extending proximally toward the graft bifurcation, with no apparent graft kinking (Figure 1).

The subsequent day, the patient underwent a left-to-right femoro-femoral bypass using an 8 mm Dacron graft. Simultaneously, clopidogrel was discontinued, and apixaban was substituted with tinzaparin at a daily dosage of 18,000 IU, adjusted based on the patient's body weight (therapeutic dose). The patient was discharged five days after the procedure fully motile with no symptoms of ischemia and a functioning bypass graft.

After discharge, the patient was maintained on in-hospital anticoagulation therapy and was referred to consult with an allergologist (unavailable in our hospital) for a thorough investigation into his apparent allergic reactions to both aspirin and clopidogrel.

**Figure 2:**
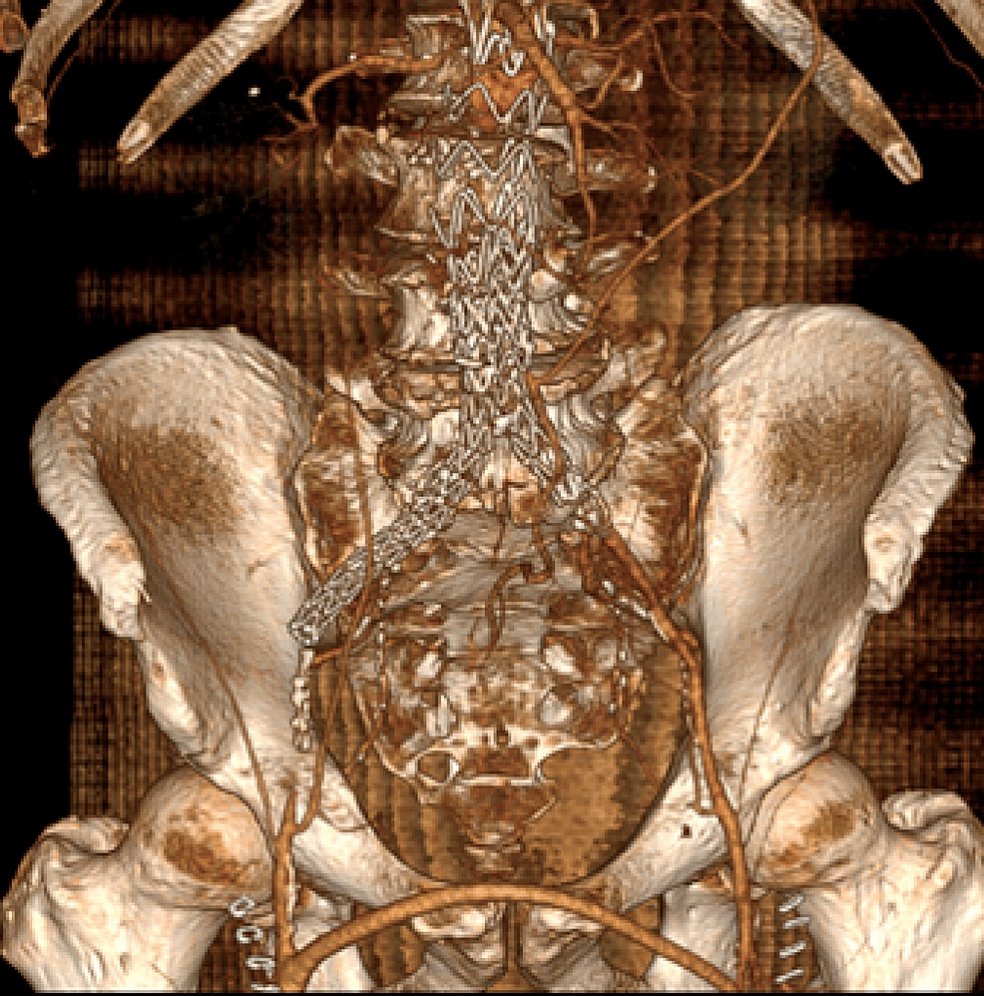
Complete Endograft Occlusion Second re-admission CT scan (3D reconstruction). Complete endograft occlusion. The left external iliac artery remains patent through collaterals from the left internal iliac artery, keeping the femoro-femoral bypass patent too.

Five days later, the patient presented once more to the emergency department with absent femoral pulses and symptoms of severe claudication. CTA examination revealed a complete endograft thrombosis and a patent femoro-femoral bypass (Figure 2).

In an urgent fashion, the patient underwent an open surgical repair with aorto-bifemoral bypass (bifurcated Dacron graft) and ligation of the femoro-femoral bypass. The patient had an uneventful postoperative course and was discharged on postoperative day 5 under apixaban treatment. At the latest follow-up, six months following the procedure, the patient remained symptom-free with a functioning bypass upon CTA examination.

## Discussion

Our recent experience urged us to conduct a literature review to explore the etiology of LGO following EVAR. We searched the PubMed library from inception up to December 2023. Subsequently, 12 studies, involving a total of 6785 patients, were included. Numerous factors were identified as contributors to LGO. The most frequently identified factors included limb extension to the EIA, highlighted in eight studies, limb graft kinking in seven studies, and limb graft oversizing exceeding 15% in five studies. Additional factors encompassed narrow EIA, iliac artery tortuosity, and iliac artery angulation, identified in four studies, whereas iliac artery calcification was implicated in three studies.

The most frequently employed devices in the included studies were Endurant (Medtronic plc, Minneapolis, MN), Excluder (W. L. Gore & Associates, Inc., Medical Products Division, Flagstaff, AZ), and Zenith (COOK MEDICAL LLC, Bloomington, IN). Additionally, Treovance (the predecessor of Treo - Bolton Medical) was utilized in a single study (Table 1).

**Table 1:** Baseline study characteristics Number of patients, factors potentially associated with LGO, and devices used in each study are listed. Factors that were present in our case are shown in bold. LGO, limb graft occlusion; CIA, common iliac artery; EIA, external iliac artery.

Study	Patients	LGO factors	Devices
Carroccio et al., 2002 [4]	351	Extension to EIA, small limb graft diameter	AneurX, Ancure, Excluder, Talent, Teramed, Vanguard
Cochennec et al., 2007 [3]	460	Limb graft kinking, first-generation devices, younger age	Zenith, Vanguard, Excluder, AneurX, EVT, Stenford, Stentor, Talent
Maleux et al., 2008 [5]	288	Limb graft kinking, extension to EIA, limb graft migration	Zenith, Excluder
Conway et al., 2012 [6]	661	Extension to EIA, iliac tortuosity, narrow EIA	Zenith, Excluder, Vanguard
Mantas et al., 2015 [7]	439	Iliac artery angulation, iliac artery calcification, limb oversizing >15%	Excluder, Zenith, Anaconda, Endurant
Taudorf et al., 2014 [8]	504	Iliac artery tortuosity	Zenith
Faure et al., 2015 [9]	1143	Extension to EIA, narrow EIA, limb graft kinking	Endurant
Moulakakis et al., 2018 [10]	579	Iliac artery angulation, iliac artery calcification, limb oversizing >15%	Excluder, Zenith, Anaconda, Endurant, Endologix
Catanese et al., 2020 [11]	276	Extension to EIA, narrow EIA, iliac artery tortuosity, iliac artery calcification, iliac artery angulation, limb oversizing >15%, limb graft kinking, narrow distal aorta	Excluder, Endurant, Treovance, Anaconda, Incraft
Bogdanovic et al., 2021 [12]	924	Narrow EIA, extension to EIA, device type	Zenith, Excluder, Endurant
Basra et al., 2024 [13]	787	Extension to EIA, limb oversizing >20%, limb graft kinking	Zenith, Endurant, Anaconda
Chacko et al., 2023 [14]	373	Narrow EIA, prior CIA stenting, extension to EIA	n/a

Our patient was diagnosed with unilateral LGO approximately one month following EVAR. According to the literature, one-third of LGOs occur within the first month following intervention. The predominant presenting symptom is intermittent claudication, as observed in our case, although presentations may range from asymptomatic to acute limb ischemia [3,15].

According to previously published reports, various risk factors have been linked to the development of LGO. Among these factors, the extension of the endograft into the EIA, along with a narrow EIA and distal aorta, as well as small aneurysmal diameters, iliac artery angulation, calcification, tortuosity, patient age, limb kinking, and oversizing >15%, as well as graft migration stand out the most [3-6,9,11-14,16-18].

While in our report several of the aforementioned risk factors can be identified such as the small size of the aneurysm (4.9 cm), the narrow EIA and distal aorta diameters of about 8 mm and 18 mm, respectively, as well as limb graft oversize exceeding 15% (we used an 11 mm limb equaling to 37.5% oversize), our patient did not display iliac vessel tortuosity or extensive calcification. Moreover, upon completion angiography, no limb kinking was observed.

Other previously identified contributing factors to LGO are small aneurysm neck, poor run-off, female gender, correction of endoleak, prior CIA stenting, chronic renal failure, arterial dissection, use of first-generation endografts or certain device types, and EVAR implemented outside instructions for use with none of these factors being present in our case [3,9,11-13,16,17].

With regard to the management of symptomatic LGO, various methods are available in the armamentarium of the treating physician. Surgical options include extra-anatomical femoro- or axillo-femoral bypasses, while endovascular therapy encompasses thrombectomy or thrombolysis with or without stent placement [19].

In our case, we managed the initial limb occlusion with an extraanatomical left-to-right femoro-femoral bypass. Thrombectomy and thrombolysis were deemed unsuitable due to potential risks of graft dislocation or hemorrhagic complications. Nevertheless, both femoro-femoral and aorto-bifemoral bypass procedures have traditionally demonstrated satisfactory patency outcomes, establishing them as our methods of choice for managing LGO following EVAR in our institution [20].

Despite the successful bypass intervention, the patient returned a few days later with bilateral limb graft thrombosis. Apparently, the narrow diameter of an aortic bifurcation and EIA are to blame. The left limb graft had neither a small diameter (15 mm) nor had been extended to the EIA. Potentially the presence of a thrombus in the previously occluded limb altered the blood flow and led to the propagation of the thrombosis to the body and consequently to the left side of the endograft. As we mentioned before, the bilateral occlusion was treated with an aorto-bifemoral bypass and ligation of the femoro-femoral graft.

Whereas according to a meta-analysis conducted by Hammond et al., unilateral LGO is observed in about 5.6% of cases following EVAR, whole-graft thrombosis is a rare event affecting approximately 13% of patients presenting with LGO [2,3,8,10,15]. Furthermore, Moulakakis et al. identified iliac angulation greater than 60°, perimeter of calcification more than 50%, and limb graft oversizing more than 15% as significant predictors for bilateral limb occlusion in their study. It is pertinent to note that these factors, like the ones described previously, were not present in our case, despite limb graft oversizing that was observed only on the right side.

The association between the patient's allergy to both aspirin and clopidogrel and the occurrence of the LGO event remains enigmatic. This ambiguity arises from the fact that the initial event transpired during the concurrent administration of apixaban and clopidogrel. Furthermore, despite the prevalence of clopidogrel resistance affecting approximately half of the individuals undergoing vascular or coronary interventions, an investigation into this possibility was not conducted at the time of the event [21].

In summary, the complex interplay of the patient's allergies, potential clopidogrel resistance, and the temporal relationship of antiplatelet administration in relation to the LGO event necessitate further investigation to unravel the contributory factors and optimize therapeutic approaches in analogous clinical scenarios [22].

## Conclusions

In conclusion, while LGO is a common complication following EVAR with the prominent contributing factor potentially being the extension of limb graft to EIA, bilateral LGO constitutes a rare phenomenon, usually being the result of diverse etiology. Antiplatelet therapy intolerance may have played a role; however, that fact remains unclear. Femoro-femoral bypass is a competent method to deal with unilateral LGO. Nonetheless, one should be aware of possible thrombus propagation and bilateral occlusion, potentially rendering extra-anatomical bypass ineffective despite its satisfactory patency rates.
